# Physical Activity in Vietnam: Estimates and Measurement Issues

**DOI:** 10.1371/journal.pone.0140941

**Published:** 2015-10-20

**Authors:** Tan Van Bui, Christopher Leigh Blizzard, Khue Ngoc Luong, Ngoc Le Van Truong, Bao Quoc Tran, Petr Otahal, Velandai Srikanth, Mark Raymond Nelson, Thuy Bich Au, Son Thai Ha, Hai Ngoc Phung, Mai Hoang Tran, Michele Callisaya, Seana Gall

**Affiliations:** 1 Menzies Institute for Medical Research, University of Tasmania, Hobart, Tasmania, Australia; 2 Can Tho University of Medicine and Pharmacy, Can Tho, Vietnam; 3 Medical Services Administration, Ministry of Health of the Socialist Republic of Vietnam, Ha Noi, Vietnam; 4 Department of Medicine, Southern Clinical School, Monash Medical Centre, Monash University, Melbourne, Victoria, Australia; Universidad Europea de Madrid, SPAIN

## Abstract

**Introduction:**

Our aims were to provide the first national estimates of physical activity (PA) for Vietnam, and to investigate issues affecting their accuracy.

**Methods:**

Measurements were made using the Global Physical Activity Questionnaire (GPAQ) on a nationally-representative sample of 14706 participants (46.5% males, response 64.1%) aged 25−64 years selected by multi-stage stratified cluster sampling.

**Results:**

Approximately 20% of Vietnamese people had no measureable PA during a typical week, but 72.9% (men) and 69.1% (women) met WHO recommendations for PA by adults for their age. On average, 52.0 (men) and 28.0 (women) Metabolic Equivalent Task (MET)-hours/week (largely from work activities) were reported. Work and total PA were higher in rural areas and varied by season. Less than 2% of respondents provided incomplete information, but an additional one-in-six provided unrealistically high values of PA. Those responsible for reporting errors included persons from rural areas and all those with unstable work patterns. Box-Cox transformation (with an appropriate constant added) was the most successful method of reducing the influence of large values, but energy-scaled values were most strongly associated with pathophysiological outcomes.

**Conclusions:**

Around seven-in-ten Vietnamese people aged 25–64 years met WHO recommendations for total PA, which was mainly from work activities and higher in rural areas. Nearly all respondents were able to report their activity using the GPAQ, but with some exaggerated values and seasonal variation in reporting. Data transformation provided plausible summary values, but energy-scaling fared best in association analyses.

## Introduction

Insufficient physical activity (PA) is a health concern in Western countries and is increasingly becoming so in the developing world [1]. Physical inactivity accounted for 9.0% of premature mortality worldwide in 2008 [1]. Monitoring population levels of total PA is important to guide the public health response to physical inactivity [2]. In addition, there is an interest in tracking trends within specific domains. The occupational domain is of particular interest in countries experiencing a shift from physically active occupations such as farming and forestry toward more sedentary, office-based occupations [3, 4]. Other domains to warrant attention are transportation and discretionary activities, with sedentary activity a recent focus of attention [5].

The Global Physical Activity Questionnaire (GPAQ) is used for surveillance of risk factors for non-communicable disease (NCD) in member countries of the World Health Organization (WHO). GPAQ was developed after a review of available tools and in consultation with experts [6]. It was intended as an improvement on the International Physical Activity Questionnaire (IPAQ), but its reliability and validity for use in cross-country comparisons has been found to be no better than that of IPAQ [7]. What has not been provided to users of GPAQ is adequate guidance in the use, interpretation and reporting of the information collected. This is a short-coming, because there are specific issues that arise in the administration of a PA questionnaire in developing countries. These include lower levels of literacy, non-familiarity with Western concepts of intensity of effort, and unstable work patterns [8] conditioned on seasonal cycles in rural areas [9]. Irrespective of country of application, there are reporting issues that arise because the data are zero-inflated and right-skewed. The GPAQ Analysis Guide [10] provides limited guidance in these respects.

The first aim of this study was to provide the first national estimates of PA for Vietnam. Our second aim was to investigate issues arising in the handling of the data that could have bearing on the accuracy of the estimates and, where possible, to provide solutions and recommendations to assist other users of the questionnaire.

## Methods

### Study participants and sampling

The data are from a nationally-representative population-based survey of risk factors for NCD in Vietnam during 2009−10 that was designed in accordance with the WHO STEPS methodology [6]. The details have been presented previously [11]. The protocol of this survey was approved by the Ethics Committee of the Vietnam Ministry of Health and the Tasmanian Health and Medical Human Research Ethics Committee. Written informed consent was obtained from participants.

### Measurements

PA information was collected by face-to-face administration of the GPAQ. Its domains are work (paid or unpaid including study/training, household chores, harvesting food/crops, fishing or hunting for food, and seeking employment), transport (such as to travel to work, for shopping, to market, and to place of worship), and leisure. Vigorous-intensity activities were defined as “activities that require hard physical effort and cause large increases in breathing or heart rate”, and moderate-intensity activities were defined as “activities that require moderate physical effort and cause small increases in breathing or heart rate”. Local examples were depicted on visual aids (show-cards). Respondents were asked whether they engaged in these types of activities for at least 10 minutes continuously and, if so, for how many days they did so in a typical week, and for how long on a typical day. If respondents had a second type of work activity or work that varied with season or month of the year, they were asked to report also in respect of it and indicate the number of months of the year they were engaged in each type of activity. GPAQ expanded questions on sedentary behaviour (sitting or reclining in a typical day) were added to the questions on activity. Socio-demographic, other behavioural, and pathophysiological measurements including weight, height, and total fasting cholesterol were made according to the standardized STEPS procedures [6]. The questionnaire was translated into Vietnamese and back-translated to ensure the appropriate meaning of each item was retained [8].

### Data analysis

Total time spent on work, transport and leisure time activities of each intensity, weighted by GPAQ-assigned Metabolic Equivalent Task energy expenditure ratios per kilogram per hour of 4 for moderate and 8 for vigorous intensity activities, were aggregated within and over domains [6]. To supplement the information contained in the GPAQ Analysis Guide [10], details on PA coding are provided in the Supporting Information (S1 Table). In accordance with the Guide [10], WHO recommendations on PA for health were defıned as engaging in at least 150 minutes of moderate-intensity activity per week, or 75 minutes of vigorous-intensity activity per week, or an equivalent combination of moderate and vigorous intensity PA achieving at least 600 MET-minutes per week. Body mass index (BMI) was defined as weight(kgs) ÷ height(m)².

Correlation and regression analysis was used to measure associations between aggregate measures of PA for each province (e.g. the provincial proportions of persons meeting the WHO recommendations for PA) and its geographical, ethnic and climatic characteristics (including the proportion of each provincial population living in areas classified as urban) and with BMI and cardio-metabolic parameters.

Reporting errors in respect of incomplete information, implausible hours of activities (defined as reported total hours per week exceeding 16 hours of activity each day of a typical week), and/or improbable values (defined as reported values of PA requiring energy expenditure greater than average energy intake of the Vietnamese people of 2100 kcal/day [12, 13]) were identified. Log binomial regression [14] was used to compare the estimated probability of any reporting error at levels of putative explanatory factors. Four approaches to minimize the influence of large extreme values on summary (mean) estimates of PA were compared. They were transformation of the outcome variable using a Box-Cox power transformation (with a constant of 1 added to allow its use with zero values) and a shifted Box-Cox transformation (with estimation of the constant to be added that made the mean as close as possible to the median), 10% trimming (setting the weights of the largest 5% and smallest 5% of values to zero), 10% winsorizing (replacing the largest 5% of values with the value of the 95^th^ percentile and the smallest 5% of values with the value of the 5^th^ percentile), and down-sizing the largest values. Three methods of down-sizing were used. They were (a) replacing larger values of total hours per week by 7×16 hours with proportional allocation across sub-domains; (b) replacing larger values of hours per week by 7×3 hours for each domain and sub-domain with proportionate reductions across work and leisure domains [15]; and (c) replacing larger values by the level of PA requiring energy expenditure of 2100 kcal/day. All analyses were performed using complex survey methods provided by Stata version 12.0.

## Results

The study sample consisted of 14706 (53.5% female) subjects aged 25−64 years, with generally higher participation proportions among older persons. Selected characteristics of the study participants, stratified by sex and residential areas, are presented in Table 1.

**Table 1:** Characteristics of subjects^*^.

	Men		Women	
Characteristic	Urban	Rural	Urban	Rural
Age group				
25−34 years	35.6%(428/2370)	35.9%(995/4434)	35.4%(539/2823)	33.6%(1206/5079)
35−44 years	30.1%(597/2370)	30.6%(1069/4434)	28.5%(700/2823)	29.5%(1225/5079)
45−54 years	23.7%(631/2370)	22.6%(1160/4434)	23.9%(800/2823)	24.1%(1346/5079)
55−64 years	10.6%(714/2370)	10.9%(1210/4434)	12.2%(784/2823)	12.8%(1302/5079)
Ethnicity				
Kinh	95.4%(2249/2359)	93.6%(3377/4428)	95.8%(2673/2815)	94.0%(3933/5074)
Years of schooling: mean(SE)	10.4(0.1)	7.5(0.1)	9.0(0.1)	6.5(0.1)
Monthly income†				
<20 USD	8.0%(190/1900)	18.6%(1024/3903)	7.2%(238/2243)	18.4%(1241/4352)
21−40 USD	13.2%(295/1900)	24.2%(1157/3903)	14.3%(363/2243)	25.7%(1278/4352)
41−60 USD	18.8%(363/1900)	25.3%(801/3903)	19.1%(457/2243)	24.3%(865/4352)
61−80 USD	10.3%(209/1900)	7.7%(305/3903)	8.7%(219/2243)	7.9%(304/4352)
81+ USD	49.7%(843/1900)	24.1%(616/3903)	50.7%(966/2243)	23.7%(664/4352)
BMI: mean(SE)	21.9(0.1)	20.8(0.1)	21.7(0.1)	20.9(0.1)

* The data presented are mean (standard error, SE) estimated with a shifted Box-Cox power transformation, or weighted percentage (unweighted number in this category/unweighted total number).

† Monthly household income (per adult member).

Summary estimates of PA during a typical week in the past year by 25−64 year olds in the Vietnamese population are presented in Table 2. For all persons (active and inactive), the estimates are 52.0 (men) or 28.0 (women) MET-hours of PA per week. Around 70% meet the WHO recommendations for PA by adults aged 18−64 years. Overall, around 20 percent of Vietnamese people were estimated to have no activity of at least moderate intensity for at least 10 minutes at a time during a typical week. In addition, Vietnamese people were estimated to sit for 3.4 hours per day. Around three quarters do not undertake any measurable leisure-time activity. Work activities are the most common source of reported activity with 55.8% of men and 43.9% of women reporting measurable activity in that domain, whereas transport is the most common source for women with 61.7% of them reporting measurable activity. On average, active persons are estimated to accumulate 100.0 (men) and 47.2 (women) MET-hours per week (see S2 Table). Without data transformation to reduce the influence of extreme values, the estimates (see S3 Table) would be 157.7 (men) and 103.4 (women) MET-hours per week for active persons, 132.2 (men) and 89.0 (women) MET-hours per week overall, and 4.0 (men) and 4.0 (women) hours per day of sitting.

**Table 2:** Estimated proportions of Vietnamese people meeting WHO recommendations, and average time spent on physical activity (MET-hours/week) by all persons, and mean time sitting (hours/day).

	Thai Nguyen	Hoa Binh	Ha Noi	Hue	Binh Dinh	Dak Lak	HCMC	Can Tho	Total
Urban population	22.5%(0.0)	12.3%(0.0)	43.3%(0.0)	33.9%(0.0)	26.0%(0.0)	21.8%(0.0)	83.6%(0.0)	67.0%(0.0)	30.3(0.0)	
Men										
Work: mean(SE)	208.0(7.8)	177.9(14.5)	0.0(0.0)	13.0(1.6)	96.1(4.0)	160.4(6.8)	0.0(0.0)	2.3(0.3)	13.0(0.7)	
Transport: mean(SE)	14.0(1.5)	15.9(1.7)	0.0(0.0)	0.0(0.0)	1.9(0.2)	8.0(0.6)	0.0(0.0)	6.0(0.4)	0.0(0.0)	
Leisure: mean(SE)	0.0(0.0)	0.2(0.0)	0.0(0.0)	0.2(0.0)	0.0(0.0)	0.0(0.0)	0.0(0.0)	0.0(0.0)	0.0(0.0)	
Total: mean(SE)	246.2(9.0)	213.7(16.8)	27.0(3.1)	35.0(3.2)	127.5(3.8)	182.5(6.7)	14.0(1.3)	28.7(1.8)	52.0(2.0)	
Meet WHO recommendations	95.1%(925)	92.5%(504)	67.1%(746)	67.5%(542)	86.6%(1029)	92.5%(587)	51.2%(672)	69.9%(818)	72.9%(5369)	
Sedentary: mean(SE)	3.7(0.1)	3.2(0.3)	6.3(0.2)	4.0(0.1)	3.0(0.1)	2.1(0.1)	2.9(0.1)	2.0(0.1)	3.4(0.0)	
Women										
Work: mean(SE)	137.4(7.2)	145.2(21.9)	0.0(0.0)	0.7(0.1)	64.3(3.7)	98.7(6.2)	0.0(0.0)	0.0(0.0)	0.0(0.0)	
Transport: mean(SE)	13.3(0.9)	18.7(1.7)	7.4(0.4)	8.7(0.6)	9.3(0.5)	10.5(0.6)	0.0(0.0)	9.3(0.6)	8.4(0.2)	
Leisure: mean(SE)	0.0(0.0)	0.0(0.0)	0.0(0.0)	0.0(0.0)	0.0(0.0)	0.0(0.0)	0.0(0.0)	0.0(0.0)	0.4(0.0)	
Total: mean(SE)	169.1(8.2)	183.0(23.6)	30.9(1.7)	28.0(1.9)	92.3(4.0)	119.4(6.5)	7.0(0.4)	16.8(1.0)	28.0(0.8)	
Meet WHO recommendations	94.0%(817)	87.7%(593)	70.6%(439)	69.8%(803)	79.1%(910)	90.8%(859)	45.8%(721)	62.8%(495)	69.1%(6091)	
Sedentary: mean(SE)	3.6(0.1)	3.6(0.1)	5.7(0.2)	3.9(0.1)	3.0(0.1)	2.2(0.1)	3.2(0.1)	1.8(0.1)	3.3(0.0)	

Mean (standard errors, SE) estimated with a shifted Box-Cox power transformation.

**Fig 1:**
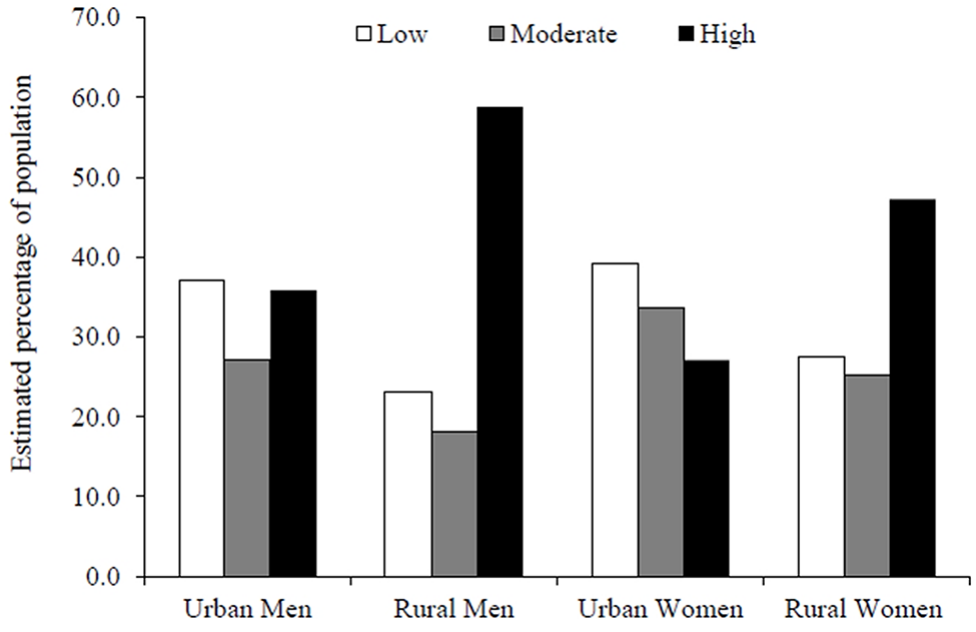
Estimated proportions of the Vietnamese population meeting the WHO recommendation of achieving at least 600 MET-minutes of activity per week (low), at least 600 MET-minutes but not 3000 MET-minutes per week (moderate), or at least 3000 MET-minutes per week (high level of activity).

Estimated proportions of the Vietnamese population meeting specified criterion values of PA are depicted in Fig 1. In rural areas, 58.8% of men and 47.3% of women had a high level of PA as defined by WHO (at least 3000 MET-minutes per week), whilst around three quarters have at least 600 MET-minutes per week. The proportions with high PA were much lower among their urban counterparts.

The percentage of Vietnamese people meeting the 75/150 WHO recommendations ranges from around 90% in the three least urbanised provinces (Hoa Binh, Dak Lak, Thai Nguyen) to around one-half (men 51.2%, women 45.8%) in HCMC, the most urbanised province of the largest city in Vietnam. The variation in proportions not active at work and overall, and in mean MET-hours at work and overall, follows a similar pattern. Residents of Ha Noi have the highest proportion of participation in leisure activity, but spend the most time sitting. These patterns of PA are replicated in urban and rural areas of each province (see S4 Table), but with time spent on each sub-domain up to 98 percent higher in rural areas than in urban areas.

The provincial proportions meeting the WHO recommendations (men r = −0.88, women r = −0.93), and of those active at work (men r = −0.91, women r = −0.93) and overall (men r = −0.86, women r = −0.84), and the provincial mean levels of PA (men r = −0.79, women r = −0.82), were each inversely associated with the provincial proportions of urban population. There were weaker associations of the aggregate PA measures with the latitude, altitude, average temperature, rainfall and proportion of minority ethnicity of each province (see S5 Table), and adjusting for the urban proportion of each province reduced each association other than those with rainfall.

The inverse associations with rainfall brought into question the seasonal timing of the survey. Overall, 92.1% (12924/14706) of respondents were interviewed in the wet season (months of the year when the average rainfall exceeds 60mm [16]). For five provinces, we were able to compare PA for those interviewed in the wet season and those in the same province interviewed in the dry season. The means were 34.7 (wet season) and 84.0 (dry season) MET-hours/week, but this ordering was reversed in the rural provinces of Hoa Binh (210 vs 140 MET-hours/week), Binh Dinh (140 vs 110 MET-hours/week) and Dak Lak (168 vs 158 MET-hours/week). The results of re-scaling the dry season values for each sub-domain to have the same median in each age, sex and urban/rural stratum as the wet season values are shown in S6 Table. The impacts were negligible on the national estimates, but of consequence for the provincial estimates for Binh Dinh where 45.6% (772/1911) of respondents were interviewed in the wet season, and Dak Lak where 88.8% (1509/1809) of interviews took place in the wet season. The median estimates of total activity were increased by 7% (Binh Dinh) and 5% (Dak Lak) for men in those provinces, and by 2% (Binh Dinh) and 0% (Dak Lak) for women.

Table 3 reports the frequency of identifiable errors in reporting PA with the GPAQ questionnaire. On its core section, 135 of the 14706 respondents failed to provide complete PA information. Another 128 respondents reported more than 7×16 = 112 hours of activity per typical 7-day week, and 2395 other respondents reported levels of usual activity that improbably required energy expenditure every day of a typical week in excess of the average energy intake (2100 kcals) of Vietnamese people. Another 1007 respondents made errors of omission or reported unrealistically high values on two sets of added questions (those in respect of a second type of work activity and sedentary non-activity). The sum totals were 5958 errors made by 3665 different respondents.

**Table 3:** Frequency of errors (item non-response and implausible or improbable responses) in reporting physical activity with the WHO GPAQ questionnaire (N = 14706).

	Missing		Implausible‡		Improbable**	
Domains	Number*	Respondents†	Number§	Respondents†	Number¶	Respondents†
Core questions						
Balance brought forward		0		135		263
Work type 1						
Vigorous	28	28	0	135	1407	1558
Moderate	26	50	7	142	652	2184
Transport	57	104	116	251	484	2617
Leisure						
Vigorous	7	110	46	253	17	2634
Moderate	27	135	56	263	24	2658
Added questions						
Balance brought forward		2658		2931		3081
Work type 2						
Monthly allocation	55	2697	0		0	
Vigorous	7	2701	499	3078	227	3262
Moderate	8	2708	52	3078	547	3540
Sedentary	249	2931	515	3081	845	3665

* Number of item non-responses.

† Cumulative number of different respondents.

‡ Reported physical activity ˃112 hours per week (7 days × 16 hours/day), in near accordance with Global Physical Activity Questionnaire Analysis Guide.

§ Number of item non-responses and implausible values.

¶ Number of item non-responses, implausible values and improbable values.

**Energy expenditure from reported activity > average energy intake per day of Vietnamese people (2100 kcal).

The 2954 reporting errors made by 2658 persons on core questions were more frequently made by men and particularly the less well-educated among them, younger persons, all those who reported a second type of work activity and residents of rural areas who did not (interaction p<0.001), those of non-Kinh ethnicity, and persons from low-income households (see S7 Table).

The option to report a second type of activity was taken up by 840 respondents (465 men and 375 women) of whom 87.6% (720/840) were from rural areas. The average time they spent on each activity was 7.1 (SD 2.3) months for the first activity and 4.9 (SD 2.2) for the second activity. The work PA levels of respondents who reported a second activity were 67.3% higher (335.9 vs 109.7 MET-hours/week) than of those without a second type of activity, with total PA that was 60.1% (357.3 vs 140.9 MET-hours/week) higher. The most common types of second activity (see S8 Table) were farming, construction (men), and house-keeping (women).

Estimates of self-reported time spent on PA made with alternative approaches of minimising the influence of large values are presented for men and women in Table 4. The shifted Box-Cox transformation of these data produced mean estimates that most closely approximated the weighted median values (the zero values of PA required a constant to be added, and the constant was chosen to ensure this). A Box-Cox transformation with an added constant of 1 produced estimates that were comparable but generally less accurate (87.4, 48.1, 47.3 and 26.9 MET-hours/week by active men, all men, active women and all women respectively). Of the other methods, down-sizing category totals according to the IPAQ guidelines (maximum 7×3 hours/week) provided summary values that were most comparable to data transformation. Each of the methods was more successfully applied to the data for active persons (no zero values) than to the data for all persons. The results were similar for urban and rural areas (data not shown).

**Table 4:** Estimates of self-reported time spent on physical activity (MET-hours/week) made with alternative approaches to reducing the influence of improbably and/or implausibly large values.

			Mean (SE)*						
Domains	Median (IQR)†	As measured		Box-Cox transformed‡	10% trimmed§	10% winsorized¶	Down-sized (total hours)**	Down-sized (category hours)††	Down-sized (energy expenditure)‡‡
Active men									
Work activity	148.0(80.0,224.0)	179.1(5.5)		133.8(4.0)	162.9(4.5)	170.4(4.8)	178.8(5.5)	84.5(2.1)	150.9(3.9)
Transport	19.7(12.7,30.3)	34.6(2.6)		19.7(1.0)	27.9(0.9)	31.1(1.2)	34.5(2.6)	29.0(1.0)	30.7(1.9)
Leisure activity	17.3(11.0,28.0)	24.9(0.0)		17.2(0.0)	21.0(0.0)	22.7(0.0)	24.8(0.0)	13.0(0.0)	23.2(0.0)
Total activity	100.0(39.0,192.7)	157.7(4.1)		100.0(2.4)	139.4(3.2)	149.5(3.6)	157.3(4.1)	81.7(1.9)	133.5(2.9)
All men									
Work activity	13.0(0.0,132.0)	110.1(3.5)		13.0(0.7)	90.5(2.7)	102.0(3.1)	109.9(3.5)	51.2(1.5)	91.5(2.6)
Transport	0.0(0.0,9.3)	15.5(0.7)		0.0(0.0)	10.6(0.4)	13.7(0.5)	15.5(0.7)	13.7(0.5)	13.8(0.6)
Leisure activity	0.0(0.0,0.0)	6.4(0.4)		0.0(0.0)	3.6(0.2)	4.8(0.2)	6.4(0.4)	3.4(0.2)	6.0(0.3)
Total activity	52.0(18.7,152.0)	132.2(3.6)		52.0(2.0)	113.3(2.9)	123.6(3.2)	131.9(3.6)	68.4(1.6)	111.4(2.7)
Active women									
Work activity	98.0(42.7,168.0)	126.4(3.8)		85.3(2.5)	110.5(3.1)	119.4(3.5)	126.1(3.8)	73.7(2.1)	115.2(3.2)
Transport	18.7(14.0,28.0)	29.5(0.9)		18.7(0.5)	24.7(0.6)	26.5(0.6)	29.4(0.9)	26.5(0.6)	28.0(0.8)
Leisure activity	14.0(10.7,24.0)	20.4(0.0)		14.3(0.0)	17.6(0.0)	18.8(0.0)	20.3(0.0)	14.1(0.0)	19.5(0.0)
Total activity	42.0(24.0,112.0)	103.4(2.5)		47.2(1.1)	85.6(1.7)	95.2(2.0)	103.2(2.5)	66.6(1.2)	94.3(2.0)
All women									
Work activity	0.0(0.0,50.7)	66.2(2.2)		0.0(0.0)	47.8(1.3)	57.3(1.6)	66.1(2.2)	36.9(1.0)	59.0(1.8)
Transport	9.3(0.0,14.0)	18.4(0.5)		8.4(0.2)	13.8(0.3)	16.6(0.4)	18.3(0.5)	16.6(0.4)	17.5(0.5)
Leisure activity	0.0(0.0,0.0)	4.4(0.3)		0.4(0.0)	2.4(0.1)	3.4(0.2)	4.4(0.3)	3.1(0.2)	4.2(0.2)
Total activity	28.0(13.7,92.0)	89.0(2.3)		28.0(0.8)	70.4(1.4)	80.4(1.8)	88.8(2.3)	56.6(1.1)	80.7(1.8)

* Mean (standard error, SE) estimated by the Hansen-Hurwitz estimator for cluster survey designs with clusters sampled with unequal probabilities and with replacement.

† Median (interquartile range).

‡ Summary values estimated with a shifted Box-Cox transformation.

§ Top 5% and bottom 5% distribution set to missing, and non-missing data reweighted.

¶ Top 5% and bottom 5% of distribution reset to 95^th^ and 5^th^ percentiles respectively.

** Total hours per week reset to 7×16 hours if in excess of 7×16 hours (this allows a person to work more than 16 hours per day on some days of the week) with proportional allocation across sub-domains.

†† Total transport, total moderate–intensity activity (work and leisure) and total vigorous-intensity activity (work and leisure) per week each reset to 7×3 hours if in excess of 7×3 hours, with proportionate reductions across work and leisure domains.

‡‡ Reported values set to the level of physical activity requiring energy expenditure of 2100 kcal/day.

Table 5 shows that work activity and total PA were negatively correlated with BMI, and transport activity more weakly so, while leisure-time activity was positively correlated with BMI. The strongest correlations between time spent on work activity (or total PA) and BMI were produced by the energy-scaling method consistently across strata of sex and activity and within domains of strata. The associations are generally similar when stratified by urban/rural residence (data not shown). These findings were replicated with fasting total cholesterol (data not shown).

**Table 5:** Association of estimates of self-reported time spent on physical activity (MET-hours/week), made with alternative approaches to reducing the influence of improbably and/or implausibly large values, with BMI.

	Pearson product-moment correlations							
Subjects and domains	As measured	Box-Cox transformed†	10% trimmed‡	10% winsorized§	Down-sized (total hours)¶	Down-sized (category hours)††	Down-sized (energy expenditure)‡‡	Rank correlation
Active men								
Work activity	–0.110***	–0.111***	–0.092***	–0.112***	–0.112***	–0.065***	–0.157***	–0.111***
Transport	0.017	–0.017	–0.012	–0.009	0.016	–0.012	–0.001	–0.018
Leisure activity	0.057*	0.056*	0.059***	0.082**	0.061*	0.017	0.062*	0.058*
Total activity	–0.103***	–0.108***	–0.086***	–0.104***	–0.105***	–0.085***	–0.143***	–0.112***
All men								
Work activity	–0.101***	–0.097***	–0.090***	–0.103***	–0.103***	–0.086***	–0.133***	–0.102***
Transport	–0.046***	–0.074***	–0.053***	–0.066***	–0.047***	–0.066***	–0.060***	–0.075***
Leisure activity	0.079***	0.103***	0.096***	0.105***	0.080***	0.080***	0.086***	0.106***
Total activity	–0.097***	–0.092***	–0.086***	–0.099***	–0.099***	–0.090***	–0.133***	–0.093***
Active women								
Work activity	–0.062***	–0.064***	–0.041***	–0.066***	–0.062***	–0.035*	–0.097***	–0.059***
Transport	–0.014	0.001	–0.024**	–0.016	–0.015	–0.016	–0.030*	–0.007
Leisure activity	0.154***	0.218***	0.156***	0.205***	0.168***	0.144***	0.200***	0.223***
Total activity	–0.074***	–0.058***	–0.092***	–0.084***	–0.075***	–0.071***	–0.106***	–0.072***
All women								
Work activity	–0.095***	–0.116***	–0.101***	–0.108***	–0.095***	–0.098***	–0.125***	–0.120***
Transport	–0.035*	–0.050***	–0.049***	–0.045***	–0.035*	–0.045***	–0.046***	–0.056***
Leisure activity	0.147***	0.140***	0.126***	0.166***	0.153***	0.144***	0.161***	0.169***
Total activity	–0.083***	–0.077***	–0.084***	–0.094***	–0.084***	–0.085***	–0.114***	–0.082***

* p<0.05

**p<0.01

***p<0.001, all model were adjusted for age, years of education, smoking, and alcohol consumption.

† Summary values estimated with a shifted Box-Cox transformation.

‡ Top 5% and bottom 5% distribution set to missing, and non-missing data reweighted.

§ Top 5% and bottom 5% of distribution reset to 95^th^ and 5^th^ percentiles respectively.

¶ Total hours per week reset to 7×16 hours if in excess of 7×16 hours (this allows a person to work more than 16 hours per day on some days of the week) with proportional allocation across sub-domains.

†† Total transport, total moderate-intensity activity (work and leisure) and total vigorous-intensity activity (work and leisure) per week each reset to 7×3 hours if in excess of 7×3 hours, with proportionate reductions across work and leisure domains.

‡‡ Reported values set to the level of physical activity requiring energy expenditure of 2100 kcal/day.

## Discussion

Around 70 percent of Vietnamese persons aged 25−64 years meet the WHO recommendations of PA for health, and around 20 percent had no activity that required at least small increases in breathing or heart rate over a period of at least 10 minutes during a typical week. On average, reported activity was 52.0 MET-hours (men) or 28.0 MET-hours (women). Consistent with previous local surveys [17, 18] and recent studies in both developing and developed nations [19–22], work activities contributed the vast bulk of recorded activity. Measurable travel takes up 18 minutes/day for the average Vietnamese woman, but almost no time for the average Vietnamese man, and three quarters undertake no leisure activities that are measurable by these methods. Work and total activity were significantly higher in less urbanised provinces and in the rural areas of each province.

Our estimated proportions of Vietnamese people meeting the WHO recommendations are similar to those from a previous survey in Vietnam [23]. They are also similar to the results of pooled analyses of GPAQ survey results in five Asian countries [20] and 22 African countries [19]. The first used an old version of the WHO recommendation that, if applied in our study, would have reduced the proportions by 3.3 (men) or 2.5 (women) percentage points. The pooled analysis of 51 mainly developing countries produced higher estimates using the IPAQ questionnaire, but overestimation of PA by IPAQ has been identified previously [24–26]. The common feature of the three pooled analyses is the heterogeneity in the country-specific estimates that was attributed to the timing of the surveys given seasonal patterns of agricultural activities [20, 21], differences in the culture and religion [19, 20], and reporting errors [19–21]. Between-country differences in urbanization were speculated [19–21] to be a possible contributor. We too found considerable variation in PA, but between the provinces of a single country, and the strongest predictor of that variation–explaining 60 to 86 percent–was the urban population proportion of each province. Consistent with this, analyses of national survey data from China during 1991–2006 [3] showed that more than four-fifths of the decline in occupational PA for men, and nearly two-thirds of the decline for women, were predicted by factors associated with urbanization. Occupational PA comprises a major portion of total PA in Vietnam and, unless PA in other domains (transport and leisure) can be increased to compensate, overall PA will decline if occupational PA diminishes in response to further industrialization.

The second aim of this study was to investigate issues arising in the use of the GPAQ instrument and in analysis of the data collected that could influence the accuracy of the estimates. A recent assessment [7] is that the GPAQ has only poor to fair criterion validity but nonetheless was considered a suitable and acceptable instrument for monitoring the PA of populations. Similar conclusions specifically for the Vietnamese population were reached in a study [9] conducted in the highly urbanised province of HCMC, even though the validity of the instrument in rural Vietnam, where 70% of the population lives and educational standards are lower [11], is untested.

The HCMC study provided the important caveat that seasonal PA differences between the wet and dry seasons have to be taken into account. We found that reported PA levels were higher in the dry season in urbanised provinces, but the reverse was the case in rural provinces where the wet season coincides with harvest time and requires high activity levels irrespective of the conditions. GPAQ seeks reporting of PA in a typical day of a typical week, but these and other [20, 21] results suggest respondents in developing countries are unduly influenced in reporting by their most recent activity. Adjusting for the wet/dry differences made almost no difference overall, but the provincial estimates were decreased in more urbanised provinces and increased in less urbanised provinces.

In relation to other complex constructs of GPAQ, 98% of respondents were able to complete the interviewer-administered questionnaire but around one-in-six reported unrealistically large values. Over-estimation of self-reported PA in response to the GPAQ instrument when administered in the Vietnamese population has been described previously [9]. In our study, most respondents who did not complete the questionnaire or provided exaggerated values were those from rural areas where educational levels are lowest, and familiarity with Western concepts of intensity and continuity of effort would be least. Our group [8] identified that seasonal stability of work patterns influenced the reporting of PA by GPAQ in a study conducted in Can Tho province, and we modified GPAQ for use in this study by allowing respondents to report a second type of work activity. Only around 6% of the sample did so, but more than 80% of those who did were from rural areas. Reporting errors were most common among rural respondents and all those who reported a second work activity. This was independent of education levels, suggesting that work activities in the rural setting are difficult to report accurately and that unstable work patterns add to the difficulty irrespective of urban/rural location.

Of the several methods for handling the zero-inflated and right-skewed data, the shifted Box-Cox transformations produced the most plausible summary values of PA. For data with zero values, a Box-Cox transformation requires a constant to be added to each observation, and we added the value that produced a design-based mean most like the corresponding median in each stratum and sub-domain. Searching for this value was straightforward and feasible to do. A Box-Cox transformation with a constant of 1 added produced comparable but generally less accurate results.

Significantly protective associations were observed between work and transport activity and NCD risk factors including body size/fatness and cholesterol. These findings are biologically plausible and underline the potential importance of work-related sources of PA in preventing NCD in this population. In contrast to previous findings in developed populations [27], but consistent with that of a previous investigation conducted in the Chinese population [28], leisure-time activity was positively associated with body size/fatness (or cholesterol), even after adjusting for a number of potential confounding factors. That leisure-time activity was most common among well-educated and high income persons living in urban areas, who were less active in other domains (work and transport), may provide the explanation. Interestingly, whilst the shifted Box-Cox transformations provided the most plausible summary estimates, the strongest correlations were produced by energy-scaled values that reduced reported PA to maximum values more consistent with average energy intake in the Vietnamese population.

The present investigation has several strengths. First, the data were collected from a nationally-representative survey of the Vietnamese population. The large sample and the comprehensive measurements of PA across all domains allowed analyses stratified by sex and rural/urban location. The availability of data on other behavioural risk factors for NCD made it possible to take account of putative confounding and mediating factors. The interviews were conducted by trained staff in accordance with standardised protocols [6] designed to minimise avoidable sources of random error and bias, and using a culturally-sensitive instrument that had been translated and back-translated. The GPAQ instrument had been tested for use in the Vietnamese population [8, 9], and modified by us to take account of some of its shortcomings [8].

However, our study has limitations. Whilst the response proportion was high for a study requiring lengthy clinic attendance with overnight fasting and blood-sampling, the possibility of non-participation bias cannot be discounted. Secondly, measurements by GPAQ are acknowledged [7–9] to be subject to very substantial error. Measurement of PA by more accurate and objective devices such as motion sensors would be an improvement, but such methods are infeasible for large-scale field work in many low resource countries including Vietnam. Furthermore, we did not measure some important risk factors for NCD including total energy intake, and failing to adjust for such factors may have influenced the findings.

## Conclusions

In conclusion, seven-in-ten Vietnamese people aged 25–64 years meet WHO recommendations for total PA, which was mainly from work activities and higher in rural areas. Nearly all respondents were able to report their activity in response to GPAQ, but with some exaggerated values and seasonal variation in reporting. Data transformation provides plausible summary values, but energy-scaling fared best in association analyses.

## Supporting Information

S1 Table Coding rules for GPAQ physical activity data*. (DOCX)

S2 Table Estimated proportions of Vietnamese people with recorded activity, and average time spent on physical activity (MET-hours/week) by those with recorded activity. (DOCX)

S3 Table Average time spent on physical activity (MET-hours/week) by those with recorded activity and by all persons, and mean time sitting (hours/day). (DOCX)

S4 Table Estimated proportions of Vietnamese people without recorded activity, meeting WHO recommendations, and average time spent on physical activity (MET-hours/week) by those with recorded activity and by all persons, and mean time sitting (hours/day). (DOCX)

S5 Table Correlations of the provincial proportion of inactive persons and provincial mean values* of physical activity for the work domain and overall with the provincial proportions of urban population and minority ethnicity, and the average annual rainfall, latitude, altitude, and average temperature of each province. (DOCX)

S6 Table Estimates* of physical activity (MET-hours/week) without and with adjustment for seasonal variation in five provinces where measurement occurred in both wet and dry seasons, and overall (total for all eight provinces). (DOCX)

S7 Table Factors associated with the number of errors (item non-response and implausible, or improbable responses) in reporting physical activity in response to core questions in GPAQ. (DOCX)

S8 Table Summary of the work activities of respondents who reported having two types of work activities. (DOCX)
