# Supplementary material for: Physical Activity in Vietnam: Estimates and Measurement Issues
Source: PLoS One. 2015 Oct 20;10(10):e0140941. doi: 10.1371/journal.pone.0140941 (PMC4618512; doi:10.1371/journal.pone.0140941)
Supplement: S1 Table — (DOCX) [file pone.0140941.s001.docx]

| S1 Table. Coding rules for GPAQ physical activity data* | |
| --- | --- |
| No | Description |
| 1. | For vigorous intensity work activities, perform steps 2–4 with ACTIVE = P1, DAYS = P2, HOURS = P3a and MINS = P3b. |
| 2. | Replace all missing codes (77, 88, 99) and missing values (.) recorded in the DAYS, HRS and MINS fields with 0. |
| 3. | Replace ACTIVE = 1 if ACTIVE ≠ 1 and DAYS × (HRS + MINS) > 0. |
| 4. | Replace ACTIVE = 2 if ACTIVE ≠ 2 and DAYS × (HRS + MINS) = 0. |
| 5. | For moderate intensity work activities, repeat steps 2–4 with ACTIVE = P4, DAYS = P5, HOURS = P6a and MINS = P6b. |
| 6. | For travel activities, repeat steps 2–4 with ACTIVE = P7, DAYS = P8, HOURS = P9a and MINS = P9b |
| 7. | For vigorous intensity recreational activities, repeat steps 2–4 with ACTIVE = P10, DAYS = P11, HOURS = P12a and MINS = P12b. |
| 8. | For moderate intensity recreational activities, repeat steps 2–4 with ACTIVE = P13, DAYS = P14, HOURS = P15a and MINS = P15b. |
|  | If expanded question P16 is included, perform step 9 with HOURS = P16a and MINS = P16b. |
| 9. | Replace all missing codes (77, 88, 99) and missing values (.) recorded in the HRS and MINS fields with 0. |
|  | If an optional second type of work activity is included with questions P1–P6 referring to work type 1 and xP1–xP6 referring to work type 2, and additional questions xP0a and xP0b referring to the months of work of type 1 and type 2 respectively, perform steps 10–15: |
| 10. | For vigorous intensity work type 2 activities, repeat steps 2–4 with ACTIVE = xP1, DAYS = xP2, HOURS = xP3a and MINS = xP3b. |
| 11. | For moderate intensity work type 2 activities, repeat steps 2–4 with ACTIVE = xP4, DAYS = xP5, HOURS = xP6a and MINS = xP6b. |
| 12. | Replace all missing codes (77, 88, 99) and missing values (.) recorded in xP0a and xP0b with 0. |
| 13. | If P2 = xP2 and P3a = xP3a and P3b = xP3b and P5 = xP5 and P6a = xP6a and P6b = xP6b: review information provided on description of each type of work, and consider setting xP1 = xP4 = 2 and xP2 = xP3a = xP3b = xP5 = xP6a = xP6b = 0 if the evidence suggests these are duplicate entries. |
| 14. | If xP0a = 0 and (P1 = 1 or P4 = 1) and (xP1 = 1 or xP4 = 1): replace xP0a=6 if xP0b=0, else replace xP0a = 12 if xP0b = 12, else replace xP0a = 12 – xP0b if xP0b < 12. |
| 15. | If xP0b = 0 and (P1 = 1 or P4 = 1) and (xP1 = 1 or xP4 = 1): replace xP0b = 2 if xP0a = 12, else replace xP0b = 12 – xP0a if xP0a < 12. |
| * The coding rules assume that range checks are performed at the data entry stage to preclude entry of data for months outside the range 0–12, for days outside the range 0–7, for hours outside the range 0–24, and for minutes outside the range 0–60. | |
