# Supplementary material for: Physical Activity in Vietnam: Estimates and Measurement Issues
Source: PLoS One. 2015 Oct 20;10(10):e0140941. doi: 10.1371/journal.pone.0140941 (PMC4618512; doi:10.1371/journal.pone.0140941)
Supplement: S2 Table — (DOCX) [file pone.0140941.s002.docx]

| S2 Table. Estimated proportions of Vietnamese people with recorded activity, and average time spent on physical activity (MET-hours/week) by those with recorded activity. | | | | | | | | | | | | | | | | | | | |  |
| --- | --- | --- | --- | --- | --- | --- | --- | --- | --- | --- | --- | --- | --- | --- | --- | --- | --- | --- | --- | --- |
|  | | Thai Nguyen | | Hoa Binh | | Ha Noi | | Hue | | Binh Dinh | | Dak Lak | | HCMC | | Can Tho | | Total | |  |
| Men |  |  |  |  |  |  |  |  |  |  |  |  |  |  |  |  |  |  |  | |
| Work | % active | 90.6% | (885) | 88.3% | (252) | 41.3% | (585) | 60.4% | (351) | 68.4% | (991) | 81.6% | (278) | 27.1% | (298) | 44.6% | (659) | 55.8% | (4250) | |
|  | mean(SE) | 230.6 | (9.1) | 195.4 | (14.8) | 79.9 | (6.7) | 107.7 | (6.6) | 165.2 | (5.5) | 207.3 | (11.2) | 165.7 | (14.9) | 79.9 | (8.2) | 133.8 | (4.0) | |
| Transport | % active | 59.7% | (638) | 75.0% | (250) | 32.2% | (471) | 38.0% | (505) | 49.7% | (809) | 60.7% | (684) | 25.0% | (589) | 63.7% | (640) | 45.9% | (3643) | |
|  | mean(SE) | 39.2 | (3.0) | 24.0 | (2.7) | 17.9 | (2.1) | 14.9 | (1.2) | 24.5 | (1.7) | 19.0 | (0.9) | 21.0 | (1.7) | 14.3 | (1.3) | 19.7 | (1.0) | |
| Leisure | % active | 12.0% | (106) | 20.4% | (359) | 42.6% | (183) | 19.5% | (173) | 20.2% | (86) | 28.3% | (163) | 30.3% | (399) | 23.8% | (177) | 25.6% | (1692) | |
|  | mean(SE) | 22.0 | (4.4) | 23.3 | (6.9) | 19.5 | (1.4) | 14.0 | (0.0) | 16.1 | (1.5) | 19.3 | (2.5) | 16.0 | (1.2) | 14.0 | (1.5) | 17.2 | (0.0) | |
| Total | % active | 97.0% | (943) | 95.0% | (577) | 77.6% | (785) | 76.6% | (641) | 90.2% | (1054) | 96.1% | (754) | 59.0% | (770) | 83.0% | (873) | 80.3% | (5791) | |
|  | mean(SE) | 250.0 | (9.6) | 223.7 | (15.7) | 48.7 | (3.9) | 91.5 | (6.3) | 146.9 | (4.5) | 191.1 | (7.0) | 48.3 | (4.5) | 45.6 | (3.5) | 100.0 | (2.4) | |
| Women |  |  |  |  |  |  |  |  |  |  |  |  |  |  |  |  |  |  |  | |
| Work | % active | 90.1% | (749) | 81.9% | (519) | 37.5% | (204) | 45.1% | (705) | 64.0% | (839) | 76.1% | (712) | 9.9% | (464) | 28.9% | (104) | 43.9% | (4345) | |
|  | mean(SE) | 156.5 | (8.7) | 180.9 | (25.1) | 56.0 | (4.9) | 70.3 | (4.3) | 130.7 | (4.9) | 136.3 | (6.3) | 93.7 | (9.9) | 24.0 | (2.6) | 85.3 | (2.5) | |
| Transport | % active | 68.2% | (637) | 81.9% | (370) | 60.2% | (227) | 61.1% | (545) | 60.3% | (821) | 65.2% | (663) | 41.8% | (645) | 74.5% | (421) | 61.7% | (5272) | |
|  | mean(SE) | 28.7 | (2.0) | 27.2 | (1.5) | 16.4 | (0.8) | 18.7 | (1.0) | 23.5 | (1.2) | 21.6 | (2.2) | 14.0 | (1.4) | 15.7 | (0.8) | 18.7 | (0.5) | |
| Leisure | % active | 10.1% | (160) | 10.9% | (176) | 35.8% | (295) | 15.6% | (240) | 15.9% | (94) | 22.2% | (172) | 27.3% | (162) | 16.3% | (319) | 20.5% | (1572) | |
|  | mean(SE) | 22.7 | (0.0) | 12.9 | (0.0) | 16.2 | (1.6) | 16.5 | (1.0) | 13.2 | (1.0) | 17.2 | (2.0) | 12.5 | (0.0) | 16.3 | (1.0) | 14.3 | (0.0) | |
| Total | % active | 96.1% | (837) | 90.3% | (668) | 82.1% | (507) | 76.7% | (833) | 84.6% | (940) | 94.5% | (893) | 57.9% | (798) | 81.7% | (590) | 79.2% | (6672) | |
|  | mean(SE) | 177.6 | (8.7) | 204.6 | (25.8) | 41.5 | (2.6) | 53.3 | (3.0) | 119.5 | (4.7) | 127.7 | (6.4) | 22.9 | (1.6) | 25.3 | (1.3) | 47.2 | (1.1) | |
| Mean (standard errors, SE) estimated with a shifted Box-Cox power transformation. | | | | | | | | | | | | | | | | | | | | |
