# Supplementary material for: Physical Activity in Vietnam: Estimates and Measurement Issues
Source: PLoS One. 2015 Oct 20;10(10):e0140941. doi: 10.1371/journal.pone.0140941 (PMC4618512; doi:10.1371/journal.pone.0140941)
Supplement: S3 Table — (DOCX) [file pone.0140941.s003.docx]

| S3 Table. Average time spent on physical activity (MET-hours/week) by those with recorded activity and by all persons, and mean time sitting (hours/day) | | | | | | | | | | | | | | | | | | | |  |
| --- | --- | --- | --- | --- | --- | --- | --- | --- | --- | --- | --- | --- | --- | --- | --- | --- | --- | --- | --- | --- |
| Domain | | Thai Nguyen | | Hoa Binh | | Ha Noi | | Hue | | Binh Dinh | | Dak Lak | | HCMC | | Can Tho | | Total | | |
| Men |  |  |  |  |  |  |  |  |  |  |  |  |  |  |  |  |  |  |  | |
| Work |  |  |  |  |  |  |  |  |  |  |  |  |  |  |  |  |  |  |  | |
|  | Active: mean(SE) | 290.2 | (11.3) | 228.5 | (15.8) | 118.4 | (10.3) | 145.6 | (9.0) | 184.8 | (6.3) | 248.9 | (15.7) | 204.8 | (18.3) | 136.2 | (14.1) | 179.1 | (5.5) | |
|  | Overall: mean(SE) | 268.3 | (10.0) | 206.8 | (16.6) | 54.4 | (7.9) | 92.8 | (7.0) | 127.7 | (5.3) | 207.6 | (12.6) | 59.4 | (7.5) | 60.1 | (6.5) | 110.1 | (3.5) | |
| Transport |  |  |  |  |  |  |  |  |  |  |  |  |  |  |  |  |  |  |  | |
|  | Active: mean(SE) | 46.3 | (3.2) | 38.1 | (5.0) | 37.8 | (9.8) | 28.6 | (3.9) | 41.0 | (3.3) | 26.1 | (1.9) | 33.5 | (2.9) | 31.0 | (3.4) | 34.6 | (2.6) | |
|  | Overall: mean(SE) | 28.9 | (2.6) | 29.3 | (4.8) | 10.5 | (1.6) | 10.7 | (1.2) | 19.8 | (1.5) | 15.9 | (1.3) | 8.4 | (1.1) | 19.5 | (2.3) | 15.5 | (0.7) | |
| Leisure |  |  |  |  |  |  |  |  |  |  |  |  |  |  |  |  |  |  |  | |
|  | Active: mean(SE) | 45.6 | (0.0) | 33.6 | (0.0) | 25.9 | (0.0) | 21.1 | (0.0) | 23.1 | (0.0) | 28.3 | (0.0) | 20.3 | (0.0) | 21.7 | (0.0) | 24.9 | (0.0) | |
|  | Overall: mean(SE) | 6.9 | (1.6) | 6.4 | (1.1) | 11.1 | (1.1) | 4.1 | (0.6) | 4.9 | (0.7) | 7.8 | (1.2) | 6.3 | (0.6) | 5.4 | (0.9) | 6.4 | (0.4) | |
| Total |  |  |  |  |  |  |  |  |  |  |  |  |  |  |  |  |  |  |  | |
|  | Active: mean(SE) | 312.4 | (11.1) | 252.2 | (19.1) | 95.5 | (8.6) | 142.3 | (10.5) | 169.1 | (5.4) | 238.9 | (12.4) | 122.5 | (11.9) | 103.9 | (8.0) | 157.7 | (4.1) | |
|  | Overall: mean(SE) | 304.1 | (10.6) | 242.5 | (19.9) | 76.0 | (8.5) | 107.7 | (7.3) | 152.3 | (4.9) | 231.3 | (12.5) | 74.0 | (7.8) | 85.2 | (6.5) | 132.2 | (3.6) | |
| Sitting |  |  |  |  |  |  |  |  |  |  |  |  |  |  |  |  |  |  |  | |
|  | mean(SE) | 4.1 | (0.1) | 3.9 | (0.4) | 6.8 | (0.2) | 4.5 | (0.1) | 3.3 | (0.1) | 2.3 | (0.1) | 3.3 | (0.1) | 2.4 | (0.1) | 4.0 | (0.1) | |
| Women |  |  |  |  |  |  |  |  |  |  |  |  |  |  |  |  |  |  |  | |
| Work |  |  |  |  |  |  |  |  |  |  |  |  |  |  |  |  |  |  |  | |
|  | Active: mean(SE) | 221.3 | (11.6) | 214.4 | (29.9) | 96.4 | (9.6) | 106.3 | (8.0) | 146.8 | (5.3) | 178.1 | (10.2) | 120.6 | (10.9) | 54.8 | (5.4) | 126.4 | (3.8) | |
|  | Overall: mean(SE) | 203.1 | (11.3) | 180.8 | (29.2) | 39.9 | (4.7) | 50.8 | (4.1) | 97.4 | (5.1) | 145.5 | (10.6) | 11.9 | (1.8) | 17.0 | (2.2) | 66.2 | (2.2) | |
| Transport |  |  |  |  |  |  |  |  |  |  |  |  |  |  |  |  |  |  |  | |
|  | Active: mean(SE) | 38.3 | (2.3) | 38.5 | (3.2) | 28.1 | (2.0) | 29.8 | (1.8) | 33.2 | (1.5) | 30.7 | (4.1) | 24.5 | (3.4) | 27.6 | (1.6) | 29.5 | (0.9) | |
|  | Overall: mean(SE) | 26.1 | (1.7) | 31.7 | (2.7) | 16.7 | (1.0) | 18.8 | (1.3) | 20.0 | (1.1) | 19.6 | (2.1) | 10.4 | (1.2) | 20.8 | (1.3) | 18.4 | (0.5) | |
| Leisure |  |  |  |  |  |  |  |  |  |  |  |  |  |  |  |  |  |  |  | |
|  | Active: mean(SE) | 34.0 | (0.0) | 21.3 | (0.0) | 21.6 | (0.0) | 19.0 | (0.0) | 17.5 | (0.0) | 28.4 | (0.0) | 17.5 | (0.0) | 19.7 | (0.0) | 20.4 | (0.0) | |
|  | Overall: mean(SE) | 4.1 | (0.9) | 1.8 | (0.5) | 8.5 | (0.8) | 2.9 | (0.4) | 3.0 | (0.4) | 5.5 | (1.0) | 4.9 | (0.6) | 3.3 | (0.4) | 4.4 | (0.3) | |
| Total |  |  |  |  |  |  |  |  |  |  |  |  |  |  |  |  |  |  |  | |
|  | Active: mean(SE) | 241.5 | (12.6) | 232.8 | (30.2) | 76.4 | (5.6) | 93.3 | (5.3) | 142.0 | (5.4) | 178.5 | (11.6) | 45.4 | (3.7) | 50.5 | (2.9) | 103.4 | (2.5) | |
|  | Overall: mean(SE) | 233.4 | (11.8) | 214.3 | (28.5) | 65.1 | (4.9) | 72.6 | (4.7) | 120.5 | (5.2) | 170.6 | (11.3) | 26.8 | (2.5) | 41.1 | (2.4) | 89.0 | (2.3) | |
| Sitting |  |  |  |  |  |  |  |  |  |  |  |  |  |  |  |  |  |  |  | |
|  | mean(SE) | 4.0 | (0.1) | 4.4 | (0.1) | 6.3 | (0.2) | 4.5 | (0.1) | 3.3 | (0.1) | 2.5 | (0.1) | 3.7 | (0.1) | 2.2 | (0.1) | 4.0 | (0.1) | |
| Mean (standard error, SE) estimated by the Hansen-Hurwitz estimator for stratified cluster survey designs with clusters sampled with unequal probabilities and with replacement and persons sampled at the final stage by stratified random sampling without replacement. | | | | | | | | | | | | | | | | | | | | |
