# Supplementary material for: Physical Activity in Vietnam: Estimates and Measurement Issues
Source: PLoS One. 2015 Oct 20;10(10):e0140941. doi: 10.1371/journal.pone.0140941 (PMC4618512; doi:10.1371/journal.pone.0140941)
Supplement: S4 Table — (DOCX) [file pone.0140941.s004.docx]

| S4 Table. Estimated proportions of Vietnamese people without recorded activity, meeting WHO recommendations, and average time spent on physical activity (MET-hours/week) by those with recorded activity and by all persons, and mean time sitting (hours/day) | | | | | | | | | | | | | | | | | | | |  |
| --- | --- | --- | --- | --- | --- | --- | --- | --- | --- | --- | --- | --- | --- | --- | --- | --- | --- | --- | --- | --- |
|  | | Thai Nguyen | | Hoa Binh | | Ha Noi | | Hue | | Binh Dinh | | Dak Lak | | HCMC | | Can Tho | | Total | |  |
| Urban men |  |  |  |  |  |  |  |  |  |  |  |  |  |  |  |  |  |  |  | |
| Work | Not active | 20.9% | (32) | 45.2% | (354) | 75.3% | (87) | 48.0% | (158) | 35.5% | (44) | 32.4% | (272) | 77.3% | (419) | 57.7% | (110) | 62.4% | (1440) | |
|  | Active: mean(SE) | 147.0 | (14.9) | 118.7 | (27.3) | 53.5 | (8.9) | 86.0 | (8.7) | 141.8 | (9.4) | 148.1 | (19.8) | 158.8 | (16.8) | 80.0 | (11.6) | 114.9 | (6.0) | |
|  | Overall: mean(SE) | 105.8 | (12.9) | 3.5 | (2.2) | 0.0 | (0.0) | 2.0 | (0.5) | 68.1 | (6.5) | 60.0 | (11.0) | 0.0 | (0.0) | 0.0 | (0.0) | 0.0 | (0.0) | |
| Transport | Not active | 41.3% | (59) | 47.4% | (329) | 72.0% | (104) | 68.7% | (97) | 48.7% | (76) | 62.9% | (92) | 76.6% | (202) | 35.6% | (96) | 63.1% | (1434) | |
|  | Active: mean(SE) | 48.7 | (3.0) | 20.3 | (3.5) | 17.8 | (2.1) | 14.0 | (2.3) | 20.0 | (3.1) | 15.3 | (2.1) | 20.2 | (2.0) | 14.9 | (1.8) | 19.9 | (0.9) | |
|  | Overall: mean(SE) | 14.0 | (2.1) | 4.7 | (0.8) | 0.0 | (0.0) | 0.0 | (0.0) | 1.0 | (0.2) | 0.0 | (0.0) | 0.0 | (0.0) | 6.7 | (0.6) | 0.0 | (0.0) | |
| Leisure | Not active | 72.8% | (118) | 67.2% | (221) | 51.0% | (163) | 74.0% | (209) | 73.3% | (156) | 56.1% | (312) | 67.8% | (278) | 74.4% | (194) | 65.8% | (1507) | |
|  | Active: mean(SE) | 31.9 | (13.2) | 28.6 | (5.4) | 19.5 | (1.5) | 15.0 | (2.0) | 17.0 | (2.8) | 17.6 | (2.5) | 15.8 | (1.2) | 14.0 | (1.7) | 17.2 | (0.7) | |
|  | Overall: mean(SE) | 0.0 | (0.0) | 0.0 | (0.0) | 3.0 | (0.3) | 0.0 | (0.0) | 0.0 | (0.0) | 0.0 | (0.0) | 0.0 | (0.0) | 0.0 | (0.0) | 0.0 | (0.0) | |
| Total | Not active | 7.5% | (8) | 15.2% | (114) | 27.1% | (23) | 25.2% | (44) | 9.1% | (18) | 7.9% | (65) | 41.6% | (81) | 17.3% | (30) | 27.0% | (564) | |
|  | Active: mean(SE) | 155.9 | (18.5) | 81.0 | (12.5) | 30.0 | (1.9) | 61.0 | (8.5) | 113.0 | (8.4) | 115.0 | (13.8) | 38.2 | (4.2) | 43.6 | (4.8) | 48.0 | (2.3) | |
|  | Overall: mean(SE) | 148.0 | (14.2) | 46.0 | (14.3) | 18.0 | (1.3) | 28.0 | (3.0) | 98.0 | (7.6) | 100.1 | (11.8) | 10.5 | (1.2) | 28.7 | (2.1) | 24.5 | (1.1) | |
| Meet WHO recommendations | | 90.3% | (148) | 75.0% | (297) | 60.3% | (188) | 61.9% | (194) | 85.1% | (174) | 87.7% | (248) | 49.1% | (392) | 69.2% | (211) | 63.2% | (1568) | |
| Sedentary | mean(SE) | 4.4 | (0.2) | 4.5 | (0.4) | 7.0 | (0.3) | 4.3 | (0.2) | 2.8 | (0.1) | 2.4 | (0.2) | 3.0 | (0.1) | 1.9 | (0.1) | 3.4 | (0.1) | |
| Rural men |  |  |  |  |  |  |  |  |  |  |  |  |  |  |  |  |  |  |  | |
| Work | Not active | 6.2% | (46) | 7.2% | (131) | 46.2% | (213) | 35.3% | (248) | 30.2% | (52) | 14.6% | (395) | 51.4% | (184) | 50.9% | (256) | 36.5% | (1104) | |
|  | Active: mean(SE) | 258.0 | (12.5) | 207.8 | (16.1) | 95.9 | (10.4) | 119.2 | (9.3) | 175.2 | (7.3) | 223.6 | (12.5) | 188.0 | (31.7) | 74.8 | (8.5) | 141.4 | (5.2) | |
|  | Overall: mean(SE) | 242.4 | (9.3) | 192.0 | (18.0) | 4.0 | (1.4) | 26.1 | (3.4) | 106.1 | (4.9) | 183.3 | (7.9) | 8.0 | (2.2) | 2.7 | (0.5) | 36.1 | (2.2) | |
| Transport | Not active | 40.0% | (266) | 22.0% | (155) | 64.7% | (310) | 58.6% | (164) | 50.9% | (202) | 32.7% | (166) | 67.4% | (114) | 37.8% | (290) | 50.2% | (1720) | |
|  | Active: mean(SE) | 33.1 | (3.9) | 23.6 | (2.7) | 18.3 | (3.3) | 15.5 | (1.5) | 26.7 | (1.9) | 20.3 | (1.0) | 24.1 | (3.3) | 14.0 | (1.5) | 19.1 | (1.1) | |
|  | Overall: mean(SE) | 14.0 | (1.8) | 16.0 | (2.1) | 0.0 | (0.0) | 0.0 | (0.0) | 2.0 | (0.2) | 10.5 | (0.8) | 0.0 | (0.0) | 5.3 | (0.6) | 3.0 | (0.2) | |
| Leisure | Not active | 92.3% | (739) | 81.3% | (157) | 62.3% | (539) | 83.8% | (385) | 82.1% | (845) | 76.0% | (472) | 78.8% | (228) | 79.8% | (655) | 78.0% | (3603) | |
|  | Active: mean(SE) | 20.3 | (4.9) | 22.3 | (7.5) | 19.6 | (2.3) | 14.0 | (0.0) | 15.7 | (1.8) | 20.1 | (3.3) | 18.2 | (3.6) | 14.6 | (1.7) | 17.0 | (0.0) | |
|  | Overall: mean(SE) | 0.0 | (0.0) | 0.2 | (0.0) | 0.0 | (0.1) | 0.0 | (0.0) | 0.9 | (0.3) | 0.0 | (0.0) | 0.0 | (0.0) | 0.2 | (0.0) | 0.0 | (0.0) | |
| Total | Not active | 1.8% | (12) | 3.6% | (43) | 19.0% | (77) | 22.5% | (72) | 10.1% | (15) | 2.8% | (121) | 37.8% | (48) | 16.3% | (122) | 16.6% | (431) | |
|  | Active | 281.4 | (11.1) | 241.1 | (18.2) | 72.5 | (9.4) | 111.7 | (8.9) | 157.9 | (5.4) | 212.2 | (7.7) | 144.0 | (13.3) | 49.7 | (3.9) | 122.1 | (3.6) | |
|  | Overall: mean(SE) | 265.3 | (12.4) | 233.1 | (19.6) | 33.4 | (8.7) | 53.0 | (5.0) | 137.7 | (4.4) | 205.8 | (7.8) | 38.7 | (7.4) | 28.7 | (4.3) | 72.7 | (3.4) | |
| Meet WHO recommendations | | 96.5% | (777) | 94.9% | (207) | 72.3% | (558) | 70.3% | (348) | 87.2% | (855) | 93.8% | (339) | 61.7% | (280) | 71.2% | (607) | 77.0% | (3801) | |
| Sedentary | mean(SE) | 3.6 | (0.1) | 3.1 | (0.3) | 5.9 | (0.3) | 3.9 | (0.1) | 3.1 | (0.1) | 2.0 | (0.1) | 2.8 | (0.2) | 2.2 | (0.1) | 3.4 | (0.1) | |
| Urban women | |  |  |  |  |  |  |  |  |  |  |  |  |  |  |  |  |  |  | |
| Work | Not active | 21.0% | (56) | 51.7% | (146) | 77.7% | (554) | 65.3% | (53) | 43.0% | (70) | 37.7% | (61) | 90.0% | (205) | 72.2% | (725) | 72.0% | (1906) | |
|  | Active: mean(SE) | 94.0 | (18.8) | 97.1 | (9.9) | 56.0 | (5.9) | 42.5 | (6.9) | 85.2 | (10.8) | 86.8 | (13.7) | 87.4 | (10.7) | 21.0 | (2.9) | 60.0 | (3.1) | |
|  | Overall: mean(SE) | 56.0 | (13.5) | 0.0 | (0.2) | 0.0 | (0.0) | 0.0 | (0.0) | 13.0 | (3.6) | 42.5 | (9.9) | 0.0 | (0.0) | 0.0 | (0.0) | 0.0 | (0.0) | |
| Transport | Not active | 42.3% | (54) | 40.6% | (181) | 45.2% | (518) | 41.5% | (92) | 39.2% | (51) | 47.6% | (71) | 59.5% | (125) | 23.2% | (458) | 46.3% | (1171) | |
|  | Active: mean(SE) | 34.8 | (4.0) | 20.9 | (4.4) | 14.8 | (1.2) | 18.8 | (1.1) | 20.5 | (1.7) | 16.5 | (2.0) | 14.0 | (1.6) | 16.7 | (1.1) | 15.8 | (0.7) | |
|  | Overall: mean(SE) | 10.0 | (1.7) | 9.1 | (1.6) | 4.0 | (0.3) | 8.0 | (0.7) | 8.6 | (0.8) | 5.3 | (0.8) | 0.0 | (0.0) | 9.3 | (0.9) | 4.0 | (0.2) | |
| Leisure | Not active | 79.7% | (77) | 76.3% | (202) | 56.3% | (428) | 75.2% | (89) | 76.1% | (103) | 64.8% | (104) | 70.3% | (237) | 83.0% | (518) | 70.6% | (1902) | |
|  | Active: mean(SE) | 35.0 | (8.0) | 13.3 | (1.9) | 16.3 | (1.0) | 17.0 | (2.0) | 16.4 | (1.9) | 16.1 | (2.1) | 12.1 | (0.9) | 19.2 | (1.4) | 14.9 | (0.7) | |
|  | Overall: mean(SE) | 0.2 | (0.1) | 0.0 | (0.0) | 0.0 | (0.0) | 0.0 | (0.0) | 0.0 | (0.0) | 0.0 | (0.0) | 0.0 | (0.0) | 0.0 | (0.0) | 0.0 | (0.0) | |
| Total | Not active | 10.6% | (20) | 24.4% | (63) | 19.8% | (278) | 24.4% | (14) | 13.0% | (27) | 8.4% | (13) | 42.4% | (72) | 16.6% | (306) | 26.4% | (612) | |
|  | Active | 111.6 | (19.0) | 69.1 | (13.8) | 28.0 | (1.6) | 34.6 | (4.0) | 73.9 | (8.6) | 69.5 | (10.8) | 23.3 | (1.8) | 25.7 | (1.7) | 29.5 | (1.1) | |
|  | Overall: mean(SE) | 84.7 | (20.5) | 46.5 | (9.7) | 20.5 | (1.1) | 23.9 | (2.1) | 56.0 | (7.6) | 64.1 | (11.7) | 7.0 | (0.5) | 21.0 | (1.4) | 15.0 | (0.7) | |
| Meet WHO recommendations | | 86.0% | (86) | 69.2% | (185) | 64.8% | (346) | 67.2% | (124) | 78.6% | (106) | 84.2% | (139) | 45.1% | (216) | 64.7% | (412) | 61.0% | (1898) | |
| Sedentary | mean(SE) | 4.0 | (0.3) | 5.1 | (0.3) | 6.8 | (0.2) | 4.1 | (0.2) | 2.7 | (0.1) | 2.6 | (0.2) | 3.2 | (0.2) | 1.7 | (0.1) | 3.5 | (0.1) | |
| Rural women | |  |  |  |  |  |  |  |  |  |  |  |  |  |  |  |  |  |  | |
| Work | Not active | 6.6% | (82) | 13.3% | (188) | 50.7% | (82) | 49.5% | (114) | 33.5% | (106) | 20.0% | (164) | 90.4% | (343) | 68.7% | (142) | 49.1% | (1642) | |
|  | Active: mean(SE) | 168.0 | (7.7) | 194.0 | (29.4) | 63.9 | (7.6) | 84.1 | (4.7) | 145.4 | (4.9) | 151.6 | (7.8) | 153.5 | (18.4) | 39.5 | (4.4) | 95.5 | (3.4) | |
|  | Overall: mean(SE) | 158.4 | (7.7) | 167.6 | (27.4) | 0.7 | (0.2) | 14.0 | (1.1) | 79.2 | (4.2) | 115.3 | (7.2) | 0.0 | (0.0) | 0.0 | (0.0) | 2.5 | (0.2) | |
| Transport | Not active | 28.7% | (196) | 14.9% | (300) | 35.7% | (94) | 37.5% | (235) | 39.9% | (143) | 31.2% | (203) | 51.6% | (242) | 30.3% | (87) | 34.8% | (1447) | |
|  | Active: mean(SE) | 27.0 | (2.3) | 28.0 | (1.8) | 18.7 | (1.4) | 20.0 | (1.6) | 24.5 | (1.1) | 22.1 | (2.1) | 16.2 | (2.4) | 14.0 | (1.2) | 18.8 | (0.6) | |
|  | Overall: mean(SE) | 13.3 | (1.1) | 21.0 | (2.0) | 9.1 | (0.6) | 8.7 | (0.8) | 9.3 | (0.6) | 12.1 | (0.8) | 0.0 | (0.2) | 8.0 | (0.5) | 9.6 | (0.3) | |
| Leisure | Not active | 92.9% | (650) | 91.0% | (475) | 70.3% | (115) | 89.1% | (543) | 86.9% | (818) | 81.4% | (661) | 85.2% | (614) | 85.3% | (132) | 83.5% | (4425) | |
|  | Active: mean(SE) | 19.7 | (0.0) | 12.9 | (0.0) | 15.9 | (2.0) | 15.5 | (1.2) | 12.2 | (1.2) | 18.7 | (3.0) | 14.9 | (0.0) | 11.2 | (0.9) | 14.0 | (0.0) | |
|  | Overall: mean(SE) | 0.0 | (0.0) | 0.0 | (0.0) | 0.0 | (0.0) | 0.0 | (0.0) | 0.0 | (0.0) | 0.0 | (0.0) | 0.0 | (0.0) | 0.0 | (0.0) | 0.0 | (0.0) | |
| Total | Not active | 2.0% | (30) | 7.5% | (120) | 16.4% | (52) | 22.7% | (25) | 16.3% | (48) | 4.6% | (31) | 40.2% | (141) | 22.0% | (68) | 18.3% | (594) | |
|  | Active | 190.1 | (10.8) | 219.7 | (32.8) | 55.9 | (5.7) | 70.3 | (3.3) | 136.8 | (5.1) | 145.2 | (7.4) | 20.5 | (2.7) | 24.5 | (1.7) | 59.3 | (1.8) | |
|  | Overall: mean(SE) | 185.0 | (9.6) | 189.3 | (29.9) | 40.7 | (3.7) | 44.3 | (2.8) | 106.2 | (4.4) | 136.4 | (7.5) | 3.0 | (0.7) | 14.2 | (1.1) | 40.0 | (1.3) | |
| Meet WHO recommendations | | 96.3% | (731) | 90.4% | (408) | 75.1% | (93) | 71.1% | (679) | 79.2% | (804) | 92.7% | (720) | 49.2% | (505) | 58.6% | (83) | 72.7% | (4193) | |
| Sedentary | mean(SE) | 3.5 | (0.1) | 3.5 | (0.1) | 5.0 | (0.3) | 3.8 | (0.1) | 3.1 | (0.1) | 2.1 | (0.1) | 3.2 | (0.2) | 1.9 | (0.1) | 3.2 | (0.1) | |
| Mean (standard error, SE) estimated with a shifted Box-Cox power transformation. | | | | | | | | | | | | | | | | | | | | |
