# Supplementary material for: Physical Activity in Vietnam: Estimates and Measurement Issues
Source: PLoS One. 2015 Oct 20;10(10):e0140941. doi: 10.1371/journal.pone.0140941 (PMC4618512; doi:10.1371/journal.pone.0140941)
Supplement: S5 Table — (DOCX) [file pone.0140941.s005.docx]

| S5 Table. Correlations of the provincial proportion of inactive persons and provincial mean values* of physical activity for the work domain and overall with the provincial proportions of urban population and minority ethnicity, and the average annual rainfall, latitude, altitude, and average temperature of each province | | | | | | | | | | | | | | | | | | | | |
| --- | --- | --- | --- | --- | --- | --- | --- | --- | --- | --- | --- | --- | --- | --- | --- | --- | --- | --- | --- | --- |
|  |  | Men | | | | | |  | |  | | Women | | | | | |  | | |
|  | Work | | |  |  | Total | | |  |  | Work | | |  |  | Total | | |  |  |
|  | Inactive (%) | | MET-hours | | Inactive (%) | | MET-hours | | | Inactive (%) | | | MET-hours | | Inactive (%) | | MET-hours | | |  |
| Urban population | 0.91 | | –0.78 | | 0.86 | | –0.79 | | | 0.93 | | | –0.77 | | 0.84 | | –0.82 | | |  |
| Minority ethnicity | –0.59 | | 0.63 | | –0.47 | | 0.63 | | | –0.53 | | | 0.71 | | –0.38 | | 0.72 | | |  |
| Annual rainfall | 0.25 | | –0.41 | | 0.37 | | –0.38 | | | 0.26 | | | –0.39 | | 0.40 | | –0.34 | | |  |
| Latitude | –0.50 | | 0.45 | | –0.40 | | 0.48 | | | –0.57 | | | 0.49 | | –0.49 | | 0.57 | | |  |
| Altitude | –0.33 | | 0.37 | | –0.37 | | 0.33 | | | –0.33 | | | 0.28 | | –0.39 | | 0.23 | | |  |
| Average temperature | 0.65 | | –0.55 | | 0.65 | | –0.56 | | | 0.70 | | | –0.54 | | 0.76 | | –0.60 | | |  |
| *MET-hours per week estimated with a shifted Box-Cox transformation. | | | | | | | | | | | | | | | | | | | | |
