# Supplementary material for: Physical Activity in Vietnam: Estimates and Measurement Issues
Source: PLoS One. 2015 Oct 20;10(10):e0140941. doi: 10.1371/journal.pone.0140941 (PMC4618512; doi:10.1371/journal.pone.0140941)
Supplement: S6 Table — (DOCX) [file pone.0140941.s006.docx]

| S6 Table. Estimates* of physical activity (MET-hours/week) without and with adjustment for seasonal variation in five provinces where measurement occurred in both wet and dry seasons, and overall (total for all eight provinces) | | | | | | | | | | | | | | | | | | | | | | | | |
| --- | --- | --- | --- | --- | --- | --- | --- | --- | --- | --- | --- | --- | --- | --- | --- | --- | --- | --- | --- | --- | --- | --- | --- | --- |
|  |  | Hoa Binh | |  |  | Ha Noi | |  |  | Binh Dinh | |  |  | Dak Lak | |  |  | Can Tho | |  |  | Total | |  |
|  |  | | Adjusted | |  | | Adjusted | |  | | Adjusted | |  | | Adjusted | |  | | Adjusted | |  | | Adjusted | |
| Men |  | |  | |  | |  | |  | |  | |  | |  | |  | |  | |  | |  | |
| Work | 192.0 | | 192.0 | | 0.0 | | 0.0 | | 112.0 | | 112.0 | | 192.0 | | 192.0 | | 2.3 | | 2.7 | | 13.0 | | 13.0 | |
| Transport | 16.0 | | 16.0 | | 0.0 | | 0.0 | | 1.9 | | 1.9 | | 8.0 | | 8.0 | | 6.0 | | 6.3 | | 0.0 | | 0.0 | |
| Leisure | 0.0 | | 0.0 | | 0.0 | | 0.0 | | 0.0 | | 0.0 | | 0.0 | | 0.0 | | 0.0 | | 0.0 | | 0.0 | | 0.0 | |
| Total | 238.0 | | 238.0 | | 27.0 | | 25.8 | | 134.0 | | 144.0 | | 206.0 | | 216.0 | | 28.7 | | 28.7 | | 52.0 | | 52.0 | |
| Women |  | |  | |  | |  | |  | |  | |  | |  | |  | |  | |  | |  | |
| Work | 168.0 | | 168.0 | | 0.0 | | 0.0 | | 66.0 | | 82.8 | | 120.0 | | 120.0 | | 0.0 | | 0.0 | | 0.0 | | 0.0 | |
| Transport | 18.7 | | 18.7 | | 9.3 | | 9.3 | | 9.3 | | 9.9 | | 12.0 | | 12.0 | | 9.3 | | 9.3 | | 9.3 | | 9.3 | |
| Leisure | 0.0 | | 0.0 | | 0.0 | | 0.0 | | 0.0 | | 0.0 | | 0.0 | | 0.0 | | 0.0 | | 0.0 | | 0.0 | | 0.0 | |
| Total | 189.3 | | 189.3 | | 34.7 | | 32.0 | | 110.0 | | 112.5 | | 131.0 | | 131.0 | | 16.8 | | 15.7 | | 28.0 | | 28.0 | |
| Urban |  | |  | |  | |  | |  | |  | |  | |  | |  | |  | |  | |  | |
| Work | 3.5 | | 3.5 | | 0.0 | | 0.0 | | 48.0 | | 62.3 | | 56.0 | | 56.0 | | 0.0 | | 0.0 | | 0.0 | | 0.0 | |
| Transport | 9.3 | | 8.4 | | 0.0 | | 0.0 | | 7.5 | | 9.0 | | 4.0 | | 4.0 | | 7.5 | | 7.5 | | 0.0 | | 0.0 | |
| Leisure | 0.0 | | 0.0 | | 0.0 | | 0.0 | | 0.0 | | 0.0 | | 0.0 | | 0.0 | | 0.0 | | 0.0 | | 0.0 | | 0.0 | |
| Total | 56.0 | | 56.0 | | 18.7 | | 18.7 | | 91.0 | | 117.0 | | 105.0 | | 105.0 | | 24.0 | | 24.0 | | 21.0 | | 21.0 | |
| Rural |  | |  | |  | |  | |  | |  | |  | |  | |  | |  | |  | |  | |
| Work | 192.0 | | 192.0 | | 0.7 | | 0.7 | | 100.0 | | 106.2 | | 188.0 | | 188.0 | | 0.0 | | 0.0 | | 16.0 | | 16.0 | |
| Transport | 16.0 | | 16.0 | | 4.7 | | 4.7 | | 8.0 | | 7.6 | | 12.0 | | 12.0 | | 7.7 | | 7.7 | | 7.3 | | 7.2 | |
| Leisure | 0.0 | | 0.0 | | 0.0 | | 0.0 | | 0.0 | | 0.0 | | 0.0 | | 0.0 | | 0.0 | | 0.0 | | 0.0 | | 0.0 | |
| Total | 237.7 | | 238.0 | | 40.7 | | 40.7 | | 132.3 | | 132.3 | | 192.0 | | 196.0 | | 21.0 | | 21.0 | | 53.5 | | 52.0 | |
| *Medians of cluster medians | | | | | | | | | | | | | | | | | | | | | | | | |
