# Supplementary material for: Physical Activity in Vietnam: Estimates and Measurement Issues
Source: PLoS One. 2015 Oct 20;10(10):e0140941. doi: 10.1371/journal.pone.0140941 (PMC4618512; doi:10.1371/journal.pone.0140941)
Supplement: S7 Table — (DOCX) [file pone.0140941.s007.docx]

| S7 Table. Factors associated with the number of errors (item non-response and implausible, or improbable responses) in reporting physical activity in response to core questions in GPAQ | | | | | | | | | | | | |
| --- | --- | --- | --- | --- | --- | --- | --- | --- | --- | --- | --- | --- |
|  |  | Men | | | |  |  | Women | | | |  |
|  | % | | (n/N)* | PR | (95%CI)† | | % | | (n/N)* | PR | (95%CI)† | |
| Sex | 21.2% | | (1680/6743) | 1.00 |  | | 9.5% | | (975/7861) | 0.44 | (0.39,0.51) | |
| Age groups |  | |  |  |  | |  | |  |  |  | |
| 25−34 years | 23.4% | | (470/1415) | 1.00 |  | | 8.8% | | (266/1735) | 1.00 |  | |
| 35−44 years | 25.6% | | (525/1644) | 1.10 | (0.92,1.31) | | 12.9% | | (316/1911) | 1.48 | (1.13,1.92) | |
| 45−54 years | 16.4% | | (404/1775) | 0.70 | (0.59,0.84) | | 9.2% | | (267/2137) | 1.05 | (0.78,1.41) | |
| 55−64 years | 11.7% | | (281/1909) | 0.50 | (0.39,0.65) | | 4.0% | | (126/2078) | 0.45 | (0.32,0.64) | |
| Trend |  | |  | p<0.001 | | |  | |  | p=0.005 | | |
| Residential area |  | |  |  |  | |  | |  |  |  | |
| Urban−single job | 12.0% | | (267/2295) | 1.00 |  | | 3.8% | | (116/2753) | 1.00 |  | |
| Urban−dual job | 51.8% | | (23/60) | 4.32 | (2.83,6.59) | | 37.7% | | (22/60) | 9.80 | (5.84,16.43) | |
| Rural−single job | 22.3% | | (1181/3983) | 1.86 | (1.56,2.22) | | 10.5% | | (717/4730) | 2.72 | (2.09,3.55) | |
| Rural−dual job | 49.2% | | (209/405) | 4.10 | (3.37,4.99) | | 33.8% | | (120/316) | 8.80 | (6.35,12.17) | |
| Interaction |  | |  | p=0.004 | | |  | |  | p<0.001 | | |
| Ethnicity |  | |  |  |  | |  | |  |  |  | |
| Kinh | 19.3% | | (1112/5569) | 1.00 |  | | 8.5% | | (597/6571) | 1.00 |  | |
| Non-Kinh | 53.4% | | (567/1157) | 2.77 | (2.37,3.24) | | 27.6% | | (378/1277) | 3.26 | (2.38,4.47) | |
| P-value |  | |  | p<0.001 | | |  | |  | p<0.001 | | |
| Education levels |  | |  |  |  | |  | |  |  |  | |
| <Primary | 26.9% | | (316/1041) | 1.00 |  | | 8.2% | | (244/2157) | 1.00 |  | |
| Primary | 26.2% | | (526/1804) | 0.98 | (0.79,1.20) | | 10.9% | | (305/2097) | 1.33 | (1.00,1.76) | |
| Secondary | 21.9% | | (550/2042) | 0.81 | (0.66,1.01) | | 11.6% | | (300/1930) | 1.42 | (1.07,1.89) | |
| Senior secondary | 15.0% | | (174/936) | 0.56 | (0.42,0.75) | | 8.4% | | (77/846) | 1.03 | (0.69,1.53) | |
| College/University+ | 10.4% | | (109/901) | 0.39 | (0.29,0.52) | | 5.5% | | (48/814) | 0.67 | (0.43,1.07) | |
| Trend |  | |  | p<0.001 | | |  | |  | p=0.164 | | |
| Monthly income‡ |  | |  |  |  | |  | |  |  |  | |
| <20 USD | 32.4% | | (422/1209) | 1.00 |  | | 17.5% | | (309/1475) | 1.00 |  | |
| 20−40 USD | 23.8% | | (418/1439) | 0.74 | (0.62,0.88) | | 11.7% | | (239/1628) | 0.67 | (0.52,0.86) | |
| 41−60 USD | 22.5% | | (250/1148) | 0.70 | (0.56,0.86) | | 9.0% | | (134/1316) | 0.52 | (0.39,0.69) | |
| 61−80 USD | 19.4% | | (126/507) | 0.60 | (0.45,0.79) | | 8.3% | | (44/520) | 0.48 | (0.29,0.79) | |
| 81+ USD | 15.4% | | (247/1442) | 0.48 | (0.38,0.59) | | 6.4% | | (116/1615) | 0.37 | (0.27,0.50) | |
| Trend |  | |  | p<0.001 | | |  | |  | p<0.001 | | |
| * %(n/N): weighted percentages (unweighted number of respondents with reporting errors/total number of respondents in this category).  † PR(95 %CI): prevalence ratios (95% confidence interval).  ‡ Monthly household income per adult member. | | | | | | | | | | | | |
