# Supplementary material for: Physical Activity in Vietnam: Estimates and Measurement Issues
Source: PLoS One. 2015 Oct 20;10(10):e0140941. doi: 10.1371/journal.pone.0140941 (PMC4618512; doi:10.1371/journal.pone.0140941)
Supplement: S8 Table — (DOCX) [file pone.0140941.s008.docx]

| S8 Table. Summary of the work activities of respondents who reported having two types of work activities | | | | |
| --- | --- | --- | --- | --- |
|  | Men | | Women | |
| First type of work activity |  |  |  |  |
| Numbers of months spent per year: mean(SE)* | 7.0 | (0.1) | 7.3 | (0.1) |
| Work activity (MET-hours/week): mean(SE)* | 127.7 | (4.4) | 92.0 | (2.9) |
| Total activity (MET-hours/week): mean(SE)* | 156.6 | (4.6) | 124.7 | (3.2) |
| Job description |  |  |  |  |
| Farming | 62.0% | (315/465) | 74.0% | (281/375) |
| Selling lottery tickets | 0.7% | (4/465) | 0.0% | (0/375) |
| House keeping | 0.0% | (0/465) | 3.7% | (11/375) |
| Working in a factory | 6.0% | (15/465) | 1.9% | (7/375) |
| Selling goods (general) | 1.6% | (7/465) | 6.9% | (19/375) |
| Working as required | 4.4% | (10/465) | 0.0% | (0/375) |
| Construction | 14.5% | (48/465) | 4.3% | (13/375) |
| Feeding and taking care of animals | 5.0% | (36/465) | 4.6% | (23/375) |
| Others | 5.8% | (30/465) | 4.6% | (21/375) |
| Second type of work activity |  |  |  |  |
| Numbers of months spent per year: mean(SE)* | 5.0 | (0.1) | 4.7 | (0.1) |
| Work activity (MET-hours/week): mean(SE)* | 365.5 | (0.0) | 285.2 | (0.0) |
| Total activity (MET-hours/week): mean(SE)* | 383.7 | (0.0) | 311.0 | (0.0) |
| Job description |  |  |  |  |
| Farming | 27.5% | (142/465) | 27.0% | (98/375) |
| Selling lottery tickets | 1.2% | (6/465) | 0.0% | (0/375) |
| House keeping | 0.8% | (16/465) | 17.1% | (69/375) |
| Working in a factory | 2.4% | (7/465) | 4.6% | (11/375) |
| Selling goods (general) | 7.3% | (18/465) | 9.9% | (29/375) |
| Working as required | 12.7% | (68/465) | 10.4% | (61/375) |
| Construction | 23.8% | (107/465) | 6.3% | (21/375) |
| Feeding and taking care of animals | 11.9% | (56/465) | 13.9% | (47/375) |
| Others | 12.3% | (45/465) | 10.8% | (39/375) |
| *Mean (standard error, SE) estimated with a shifted Box-Cox transformation. | | | | |
